# Supplementary material for: Comparison of Gold Nanoparticles Deposition Methods and Their Influence on Electrochemical and Adsorption Properties of Titanium Dioxide Nanotubes
Source: Materials (Basel). 2020 Sep 25;13(19):4269. doi: 10.3390/ma13194269 (PMC7578957; doi:10.3390/ma13194269)
Supplement: Supplementary file 1 [file materials-13-04269-s001.pdf]

Supplementary Materials: Comparison of Gold Nanoparticles Deposition Methods and Their Influence on Electrochemical and Adsorption Properties of Titanium Dioxide Nanotubes

Ewa Paradowska [1,*], Katarzyna Arkusz [1] and Dorota G. Pijanowska[2]

[1] Department of Biomedical Engineering, Faculty of Mechanical Engineering, University of Zielona Gora, Prof. Zygmunta Szafrana 4 Street, 65-516 Zielona Gora, Poland; k.arkusz@ibem.uz.zgora.pl
[2] Nalecz Institute of Biocybernetics and Biomedical Engineering, Polish Academy of Sciences, Ks. Trojdena 4 Street, 02-109 Warszawa, Poland; dpijanowska@ibib.waw.pl
* Correspondence: e.paradowska@ibem.uz.zgora.pl

The Nyquist diagrams (Figure S1a) determined for TNTs (titanium dioxide nanotubes) and AuNPs (gold nanoparticles)/TNTs present fragments of incomplete semicircles. This is characteristic observation for titanium dioxide layers. Due to the fact, that nanoparticles have a diameter larger than diameter of TNTs. The value of real part of impedance for 0.1 mM AuNPs/TNTs and 1 mM AuNPs/TNTs decreases compared TNTs without Au nanoparticles. The real part of impedance values increases with the increase of the diameter of AuNPs. This is te result of blockage of TNTs trough the AuNPs with diameter of 99.4 ± 42.4 nm, and 182.3 ± 51.7 nm. The lowest imaginary part of impedance and real part of impedance was noted for AuNPs with the smallest diameter (20.3 ± 2.9 nm). The Bode diagrams presented as a Figure S1b show two time constants.

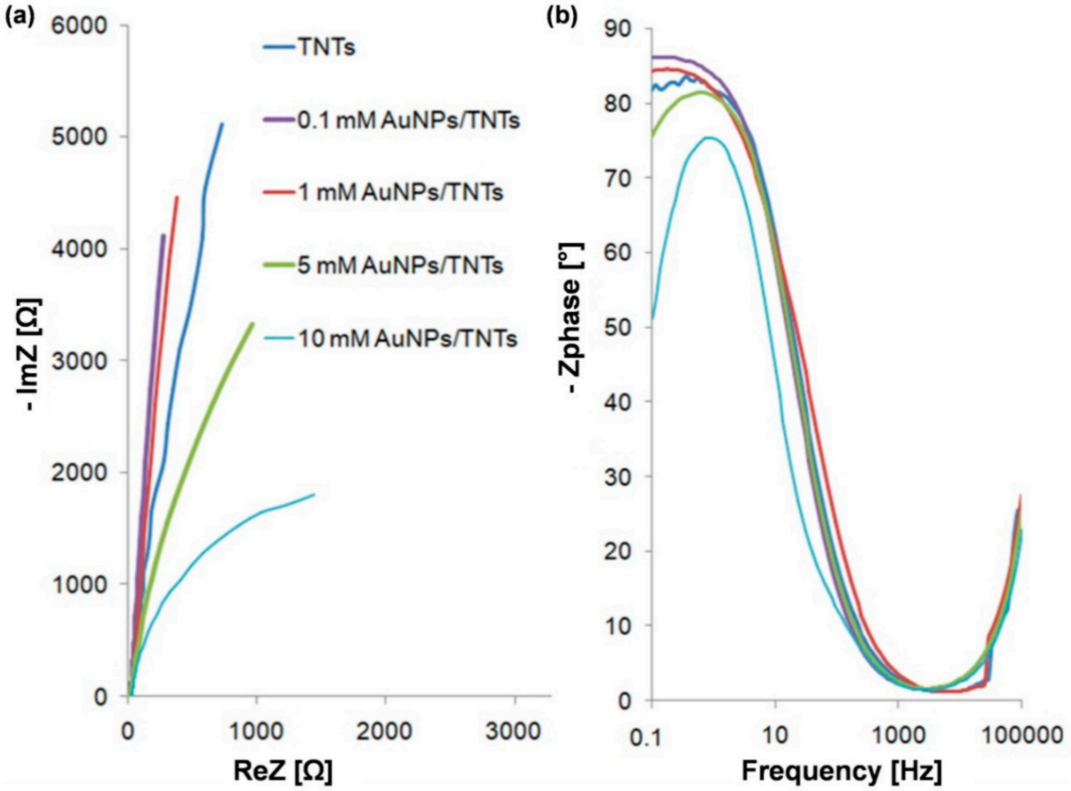

Figure 1. (**a**) Nyquist and (**b**) Bode plots for TNTs and AuNPs/TNTs deposited using cyclic voltametry method (40 cycles, 0.1 mM, 1 mM, 5 mM, 10 mM of $HAuCl_4$).

The Nyquist diagrams presented on the Figure S2a present fragments of wide semicircles. Deposition of AuNPs using CA method (carried out for 60 s) caused a decrease of the value of real part of impedance (ReZ) compared to TNTs. For longer deposition time (120 s, 180 s) the increase in the ReZ value was observed. Due to dissolving of TNTs the highest value of ReZ was observed for the deposition time of 240 s. The lowest heterogeneity (Figure S2b) have TNTs (82.8 ± 0.4°) and AuNPs for which deposition was carried out for 60 s (81.6 ± 1.0°).

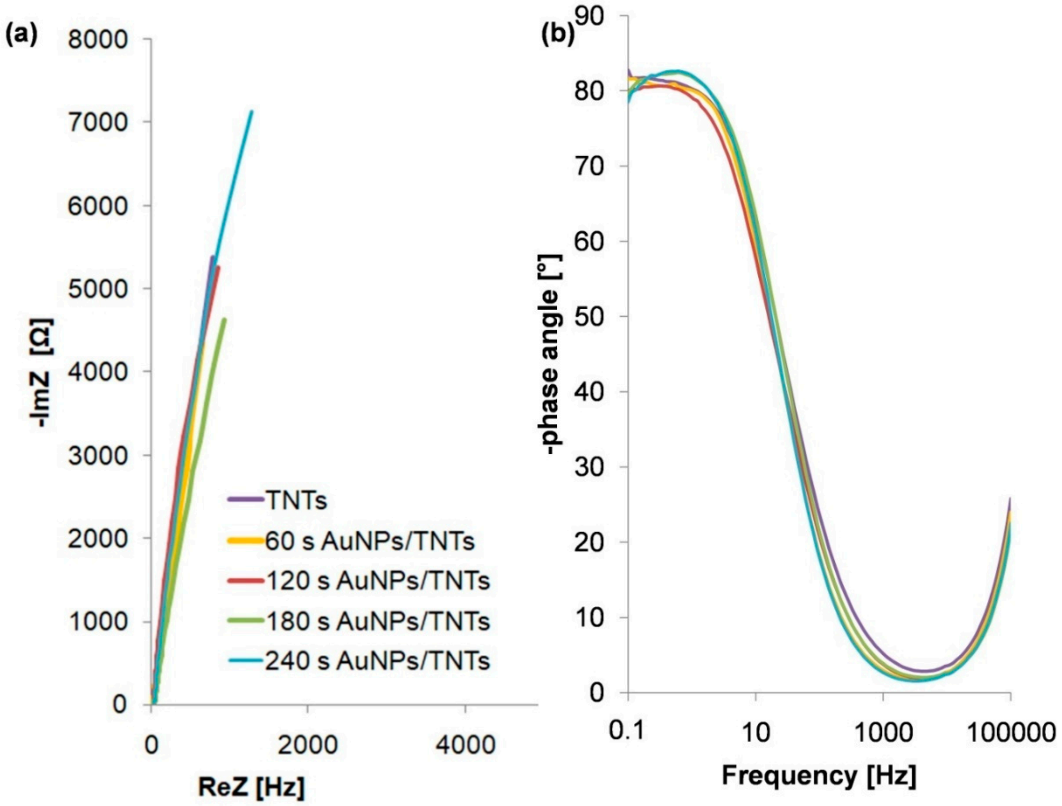

Figure S2. (**a**) Nyquist and (**b**) Bode plots for TNTs and AuNPs/TNTs deposited using chronoamperometry method for 60 s, 120 s, 180 s, 240 s—times of process.

© 2020 by the authors. Submitted for possible open access publication under the terms and conditions of the Creative Commons Attribution (CC BY) license (http://creativecommons.org/licenses/by/4.0/).
